# Synthesis of cyclic ethers by cyclodehydration of 1,*n*-diols using heteropoly acids as catalysts

**DOI:** 10.1098/rsos.180740

**Published:** 2018-09-26

**Authors:** Yufeng Sun, Yatao Huang, Minmin Li, Jia Lu, Nuo Jin, Bei Fan

**Affiliations:** Institute of Food Science and Technology, Chinese Academy of Agricultural Sciences/Key Laboratory of Agro-products Quality and Safety Control in Storage and Transport Process, Ministry of Agriculture and Rural Affairs, Beijing 100193, People's Republic of China

**Keywords:** heteropoly acid, cyclic ether, cyclodehydration, catalyst, synthesis

## Abstract

Heteropoly acids were used as catalysts for cyclodehydration of various 1,*n*-diols. Cyclodehydration of butane-1,4-diol, pentane-1,5-diol and hexane-1,6-diol catalysed by H_3_PW_12_O_40_ gave tetrahydrofuran, tetrahydropyran and oxepane, respectively. Cyclodehydration of diethylene glycol, triethylene glycol, diethylene glycol monomethyl ether and polyethylene glycol 200 catalysed by H_3_PW_12_O_40_ gave 1,4-dioxane. In particular, cyclodehydration of hexane-1,6-diol gave an excellent yield of oxepane (80%). The selectivity exhibited by the H_3_PW_12_O_40_ catalyst was even better than that exhibited by other reported catalyst systems for similar cyclodehydration reactions.

## 1. Introduction

Many natural compounds, such as inostamycins, isosorbide and polyether antibiotics, incorporate cyclic ethers as structural subunits and have significant biological activity [1–3]. Additionally, some cyclic ethers have distinctive aromas and are used as flavours [4]; (−)-ambrox is a typical representative of this type of cyclic ether [5]. Many of the commonly used synthetic approaches for the formation of cyclic ethers, including cycloaddition and cyclization, involve chlorine chemistry or heavy metals at different levels [6,7]. Additionally, cyclization reactions are often conducted under acidic conditions [8,9]. Cyclodehydration of 1,*n*-diols to cyclic ethers is an industrially important reaction [4]. These reactions are usually carried out using inorganic and organic acids, solid acid catalysts (such as clays), group (IV) metal halides, metallocenes, sulfated zirconia, zeolite or calcium phosphate. There is a strong interest in the use of solid acid catalysts to replace conventional homogeneous catalysts, such as inorganic and organic acids [10]. Although conventional catalysts are very effective, they produce highly corrosive media and chemically reactive waste streams [11]. However, using heteropoly acids (HPAs) for the general operation of large chemical processes is ecofriendly and safe [12–14].

HPA catalysts provide several advantages that make them economically and environmentally attractive [15]. HPAs can be viewed as versatile catalysts because they contain multiple active sites, including metals, protons and oxygen atoms. Protons can act as Brønsted acids to promote acid-catalysed reactions [16]. HPA catalysts can contain one or two types of acidic sites, acidic protons and/or Lewis-acidic metals [17]. Both types of acidic sites can work as active sites in acid catalysis. HPA catalysts can improve many classical acid catalysis reactions, such as cracking, condensation, isomerization, Friedel–Crafts and amination reactions [18–21]. Therefore, there has been considerable interest in the use of HPAs as catalysts.

**Figure 1.**
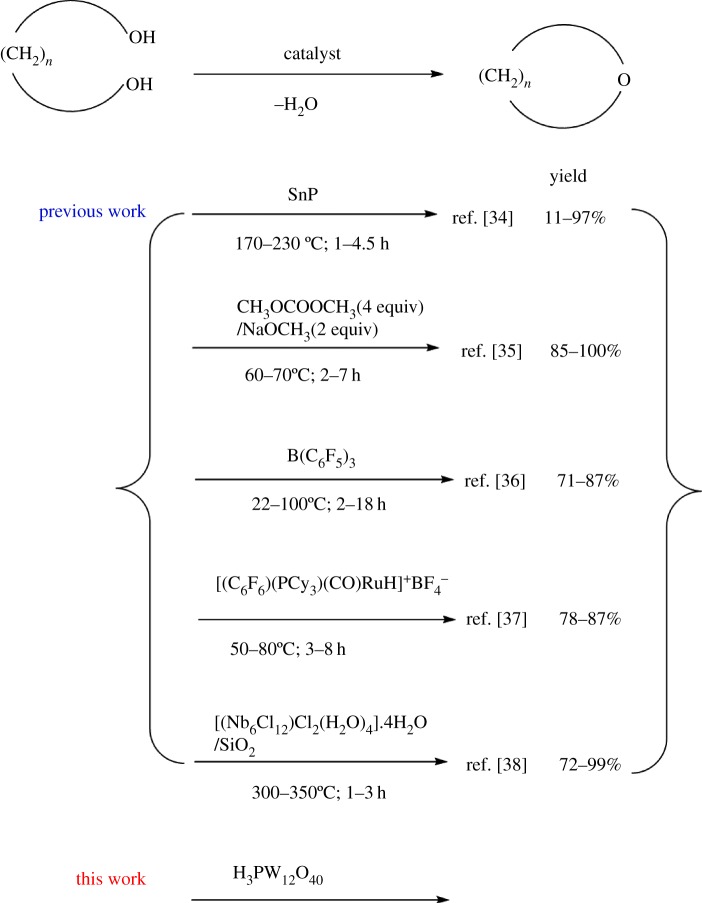
Approaches to the synthesis of cyclic ethers by cyclodehydration of 1,*n*-diols.

**Figure 2.**
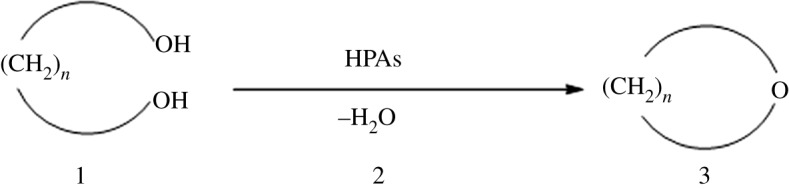
Cyclodehydration of 1,*n*-diols catalysed by HPAs.

HPAs are active and selective for tetrahydrofuran (THF) production. One commercial THF production method is based on a well-known technology that uses strongly inorganic acids as catalysts for cyclodehydration of butane-1,4-diol [22–27]. Recent advances in the synthesis of substituted THF rings have been achieved using different synthetic methods with novel catalysts and alternative starting materials [28–33]. Additionally, reports of cyclodehydration of 1,*n*-diols to cyclic ethers have been published; this is essentially a dehydration reaction between two hydroxy groups to yield an ether (figure 1) [34–38]. The present paper reports the application of HPAs as solid acid catalysts for cyclodehydration of 1,*n*-diols to their corresponding cyclic ethers with high yield and selectivity (figure 2).

## 2. Material and methods

### 2.1. Materials

All the chemicals were purchased commercially. All reagents were of analytical grade and were used directly.

### 2.2. The synthetic method of catalyst **2a–2d**

All the catalysts (**2a**–**2d**) were synthesized using the same approach. The following method is provided for catalyst **2a** as an example.

Concentrated H_3_PO_4_ was added to a boiling aqueous solution of Na_2_WO_4_·2H_2_O in a 4 : 1 acid/salt ratio and boiling was maintained in a reflux system for 8 h. The salt was precipitated by the addition of KCl, then purified by recrystallization and cooled overnight to 5°C. The product was filtered, washed and then vacuum-dried for 8 h. The product was treated with ether and a concentrated HCl (37%) solution. The released Dawson acid formed an additional compound with the ether, which allowed it to be separated from the solution. After obtaining the ether solution with the acid, the ether was eliminated and the remaining solution was placed in a vacuum-desiccator until crystallization.

### 2.3. Upscaling with **2a** as a catalyst

A mixture of butane-1,4-diol (900.12 g, 10 mol) and catalyst **2a** (49.04 g, 0.01 mol) was placed in a round-bottom flask fitted with a distillation unit. The mixture was stirred and heated in an oil bath. When the temperature reached 100°C, THF and water began to distil from the mixture. Stirring and heating at this temperature were continued until the distillation stopped (8 h). THF (664.60 g, 9.23 mol; 92.3% yield) was obtained after drying over molecular sieves and filtering.

### 2.4. Synthesis of compounds **3a–3g**

Compounds **3a–3g** were all synthesized using the same approach. The following method is provided for the synthesis of THF (**3a**) as an example.

A mixture of butane-1,4-diol (200 mmol) and an HPA (H_3_PW_12_O_40_) catalyst (0.2 mmol) was added to a round-bottom flask fitted with a distillation unit. The mixture was stirred magnetically and heated in an oil bath. When the temperature reached 100°C, THF and water began to distil from the mixture. The mixture was continuously stirred and heated at this temperature until the distillation was complete. THF was obtained by drying the distillate over CaCl_2_ and filtering. Reactions were performed with various 1,*n*-diols and the products were identified using ^1^H NMR, ^13^C NMR and mass spectrometry.

## 3. Results and discussion

The cyclodehydration reaction conditions were optimized under solvent-free conditions using butane-1,4-diol (**1a**) as the reagent.

As with all catalysis, the first step in using HPAs for selective catalytic dehydration of butane-1,4-diol to THF was to choose an appropriate HPA catalyst. The metals of the HPA catalysts were the selected focus because they are the active sites in the acid-catalysed reactions [18,19]. Accordingly, a series of HPA catalysts were prepared, as summarized in table 1, and a set of standard experimental conditions employed (100°C for 3 h) to assess the relative utility of the synthetic catalysts. The catalytic activities decreased in the following order: **2a** (H_3_PW_12_O_40_) > **2c** (H_4_SiW_12_O_40_) > **2b** (H_3_PMo_12_O_40_) > **2d** (H_4_SiMo_12_O_40_). Catalysts **2a** and **2c** provided higher yields than the other catalysts. Moreover, although Mo and W belong to the same group, they displayed different catalytic activities in this reaction; the order of the catalytic activities was in accordance with that of the Brønsted acidity of the HPAs [16]. Tungsten HPA **2a** was the catalyst of choice because of its stronger acidity, higher thermal stability and lower oxidation potential than **2b, 2c** and **2d**. Overall, catalyst **2a** (H_3_PW_12_O_40_) gave the best yield (98%).

**Table 1.** The efficiencies of various HPAs catalysts used in the synthesis of THF.

entry	catalyst	chemical composition of catalyst	yield (%)
1	**2a**	H_3_PW_12_O_40_	98
2	**2b**	H_3_PMo_12_O_40_	87
3	**2c**	H_4_SiW_12_O_40_	91
4	**2d**	H_4_SiMo_12_O_40_	82

The effect of the amount of catalyst **2a** (H_3_PW_12_O_40_) on the reaction yield was thoroughly investigated. The yield of THF (**3a**, structure shown in table 3) progressively increased from 70 to 99% with increased catalyst loading (table 2). Notably, a catalyst loading of 0.1 mol% was highly effective for the model reaction. The effect of the temperature and reaction time was subsequently investigated, both of which significantly affected the reaction. The yield of THF progressively increased from 62 to 98% with increased reaction time. A yield of 98% was obtained under optimum conditions of 0.1 mol% catalyst loading at 100°C for 3 h reaction. THF was obtained as the only product.

**Table 2.** Optimization of the synthesis of THF catalysed by H_3_PW_12_O_40_^a^.

entry	cat. loading (mmol) (%)	*T* (°C)	time (h)	yield (%)
1	0.05	100	3	70
2	0.1	100	3	98
3	0.2	100	2.5	99
4	0.1	90	10	60
5	0.1	120	3	98
6	0.1	100	1	62
7	0.1	100	2	80

^a^Reaction conditions: butane-1,4-diol (200 mmol).

**Table 3.** Cyclodehydration of 1,*n*-diols catalysed by H_3_PW_12_O_40_.

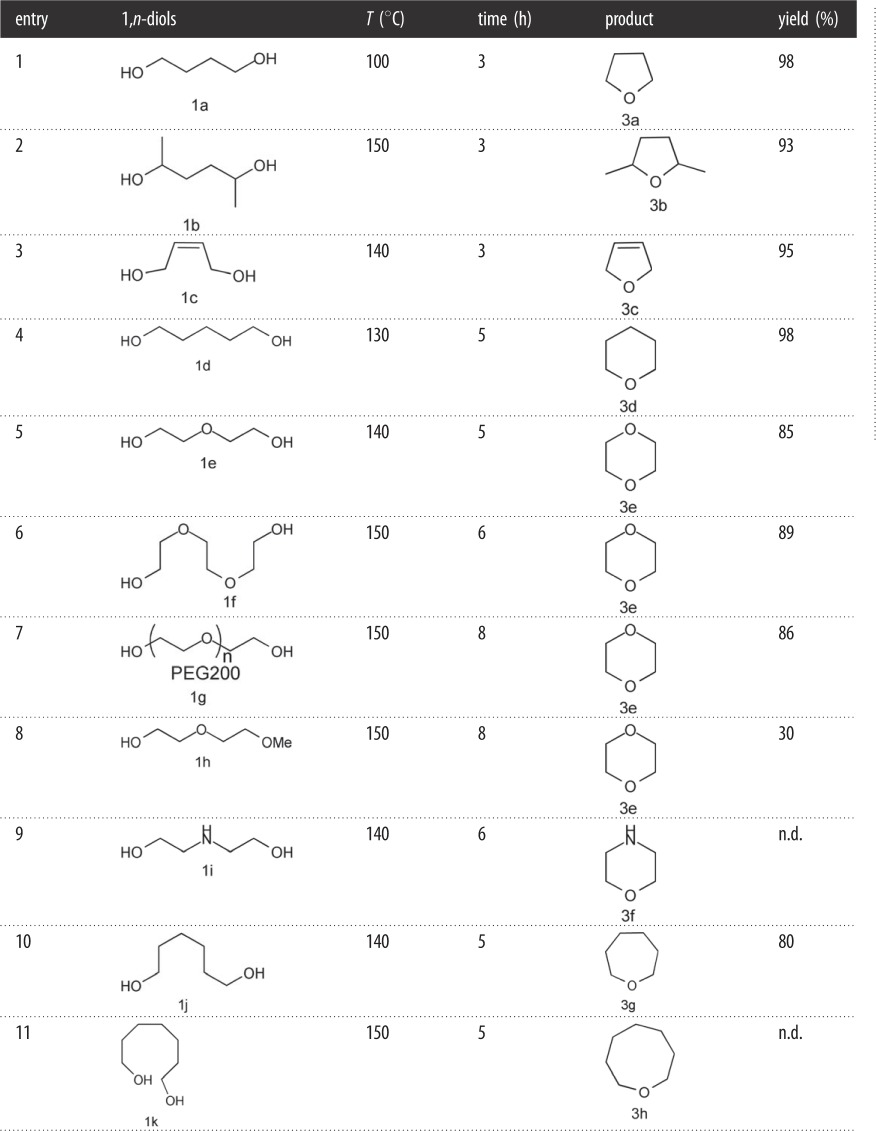

n.d.: not detected.

Based upon the above optimum reaction conditions, amplification experiments were performed. The upscaling experiment with **2a** as a catalyst was described in ‘Material and methods'. The generally good yields (664.60 g, 9.23 mol; 92.3% yield) enabled the production of THF in the order of 100 g. Therefore, this method has prospective applications in the production of THF.

Next, the optimum reaction conditions were used in the synthesis of cyclic ethers by cyclodehydration of 1,*n*-diols catalysed by catalyst **2a** (table 3). Cyclodehydration of butane-1,4-diol gave a 98% yield of THF in 3 h. As shown in table 2, cyclodehydration of hexane-2,5-diol (**1b**) required a higher temperature to afford 2,5-dimethyltetrahydrofuran (**3b**) in lower yield; (Z/E)-but-2-ene-1,4-diol (**1c**) to afford 2,5-dihydrofuran (**3c**) also required higher temperature and provided a lower yield; and pentane-1,5-diol (**1d**) gave tetrahydropyran (**3d**) in near-quantitative yield at a slightly higher temperature (table 2). As shown in table 3, diethylene glycol (**1e**) gave 1,4-dioxane (**3e**) in 85% yield, and triethylene glycol (**1f**) under these conditions gave 1,4-dioxane (**3e**) in 89% yield. These were significantly higher yields of cyclic ether **3e** from diethylene glycol (**1e**) and triethylene glycol (**1f**) than previously obtained using other catalyst systems [34–38]. Similarly, polyethylene glycol 200 (**1g**) gave 1,4-dioxane (**3e**) in an excellent yield of 86% (table 3). Cyclodehydration of diethylene glycol monomethyl ether (**1h**) and diethanolamine (**1i**) also afforded cyclic ether **3f** in slightly lower yields (table 3). Cyclodehydration of hexane-1,6-diol (**1j**) required a higher temperature and longer reaction time to yield oxepane (**3g**) in higher yield (80%; table 3) than previously obtained using other catalyst systems [34–38]. Cyclodehydration of heptane-1,7-diol (**1k**) to form **3h** did not occur because the catalyst was too far from the diol group to play a direct role.

Compounds **3a–3g** (table 3) were all synthesized using the same approach according to the synthetic method described for **3a** in ‘Material and methods’. Products **3a–3g** were identified using ^1^H NMR, ^13^C NMR and mass spectrometry.

### 3.1. Compound **3a**

^1^H NMR (300 MHz; CDCl_3_) *δ*: 3.58–3.62 (4H, m), 1.67–1.76 (4H, m). ^13^C NMR (75 MHz; CDCl_3_) *δ*: 67.6, 25.3. MS, *m/z*: 72 (M^+^).

### 3.2. Compound **3b**

^1^H NMR (300 MHz; CDCl_3_) *δ*: 4.02–4.12 (2H, m), 3.82–3.93 (2H, m), 1.98–2.02 (2H, m), 1.87–1.92 (2H, m), 1.40–1.43 (4H, m), 1.18 (6H, d, *J* = 6.3 Hz), 1.14 (6H, d, *J* = 6.3 Hz). ^13^C NMR (75 MHz; CDCl_3_) *δ*: 75.2, 74.4, 34.1, 32.9, 21.3. MS, *m/z*: 100 (M^+^).

### 3.3. Compound **3c**

^1^H NMR (300 MHz; CDCl_3_) *δ*: 5.85 (2H, s), 4.62 (4H, d, *J* = 0.6 Hz). ^13^C NMR (75 MHz; CDCl_3_) *δ*: 126.1, 75.3. MS, *m/z*: 70 (M^+^).

### 3.4. Compound **3d**

^1^H NMR (300 MHz; CDCl_3_) *δ*: 3.60 (4H, t, *J* = 5.0 Hz), 1.49–1.64 (6H, m). ^13^C NMR (75 MHz; CDCl_3_) *δ*: 68.6, 26.7, 23.3. MS, *m/z*: 86 (M^+^).

### 3.5. Compound **3e**

^1^H NMR (300 MHz; CDCl_3_) *δ*: 3.64 (4H, s). ^13^C NMR (75 MHz; CDCl_3_) *δ*: 66.9. MS, *m/z*: 88 (M^+^).

### 3.6. Compound **3g**

^1^H NMR (300 MHz; CDCl_3_) *δ*: 3.65 (4H, t, *J* = 5.7 Hz), 1.67 (4H, t, *J* = 4.2 Hz), 1.59 (4H, q, *J* = 3.0 Hz). ^13^C NMR (75 MHz; CDCl_3_) *δ*: 69.9, 30.9, 26.7. MS, *m/z*: 100 (M^+^).

## 4. Conclusion

Cyclic ethers were obtained by the reaction of 1,*n*-diols using HPA catalyst H_3_PW_12_O_40_ (**2a**), an inexpensive and simply prepared catalyst. The selectivity exhibited by catalyst H_3_PW_12_O_40_ was better than that of several reported catalyst systems for similar cyclodehydration reactions. Similar catalysts also catalysed the conversion of polyethylene glycols and triethylene glycol to 1,4-dioxane. Cyclodehydration of hexane-1,6-diol gave oxepane in excellent yield. With H_3_PW_12_O_40_ as a catalyst, the upscaling experiment provided generally good yields, enabling THF production in the order of 100 g; this method has prospective applications in the production of THF. Therefore, the HPA catalyst H_3_PW_12_O_40_ is a promising solid acid catalyst on which further research will be undertaken.

## Supplementary Material

1H NMR, 13C NMR, MS and GC spectras of products 3a–3g

## Data accessibility

The datasets supporting this article have been uploaded as part of the electronic supplementary material.
