# Supplementary material for: Synthesis of cyclic ethers by cyclodehydration of 1,n-diols using heteropoly acids as catalysts
Source: R Soc Open Sci. 2018 Sep 26;5(9):180740. doi: 10.1098/rsos.180740 (PMC6170547; doi:10.1098/rsos.180740)

**Synthesis of cyclic ethers by cyclodehydration of 1, n-diols using heteropoly acids as catalysts**

Yufeng Sun, Yatao Huang, Minmin Li, Jia Lu, Nuo Jin, Bei Fan^*^

Institute of Food Science and Technology, Chinese Academy of Agricultural Sciences / Key Laboratory of Agro-products Quality and Safety Control in Storage and Transport Process, Ministry of Agricultrue, P.R. China

^*^ E-mail: [fanbei517@163.com](mailto:fanbei517@163.com) (B. Fan). Fax: +86-10-62815969.

| **TABLE OF CONTENTS** | **PAGE** |
| --- | --- |
| 1. ^1^H NMR spectra of product 3a | S1 |
| 2. ^13^C NMR spectra of product 3a | S1 |
| 3. MS spectra of product 3a | S2 |
| 4. ^1^H NMR spectra of product 3b | S2 |
| 5. ^13^C NMR spectra of product 3b | S3 |
| 6. MS spectra of product 3b | S3 |
| 7. ^1^H NMR spectra of product 3c | S4 |
| 8. ^13^C NMR spectra of product 3c | S4 |
| 9. MS spectra of product 3c | S5 |
| 10. ^1^H NMR spectra of product 3d | S5 |
| 11. ^13^C NMR spectra of product 3d | S6 |
| 12. MS spectra of product 3d | S6 |
| 13. ^1^H NMR spectra of product 3e | S7 |
| 14. ^13^C NMR spectra of product 3e | S7 |
| 15. MS spectra of product 3e | S8 |
| 16. ^1^H NMR spectra of product 3g | S8 |
| 17. ^13^C NMR spectra of product 3g | S9 |
| 18. MS spectra of product 3g | S9 |
| 19. GC spectra of product 3a | S10 |
| 20. GC spectra of product 3b | S10 |
| 21. GC spectra of product 3c | S11 |
| 22. GC spectra of product 3d | S11 |
| 23. GC spectra of product 3e | S12 |
| 24. GC spectra of product 3g | S12 |

1. ^1^H NMR spectra of product 3a


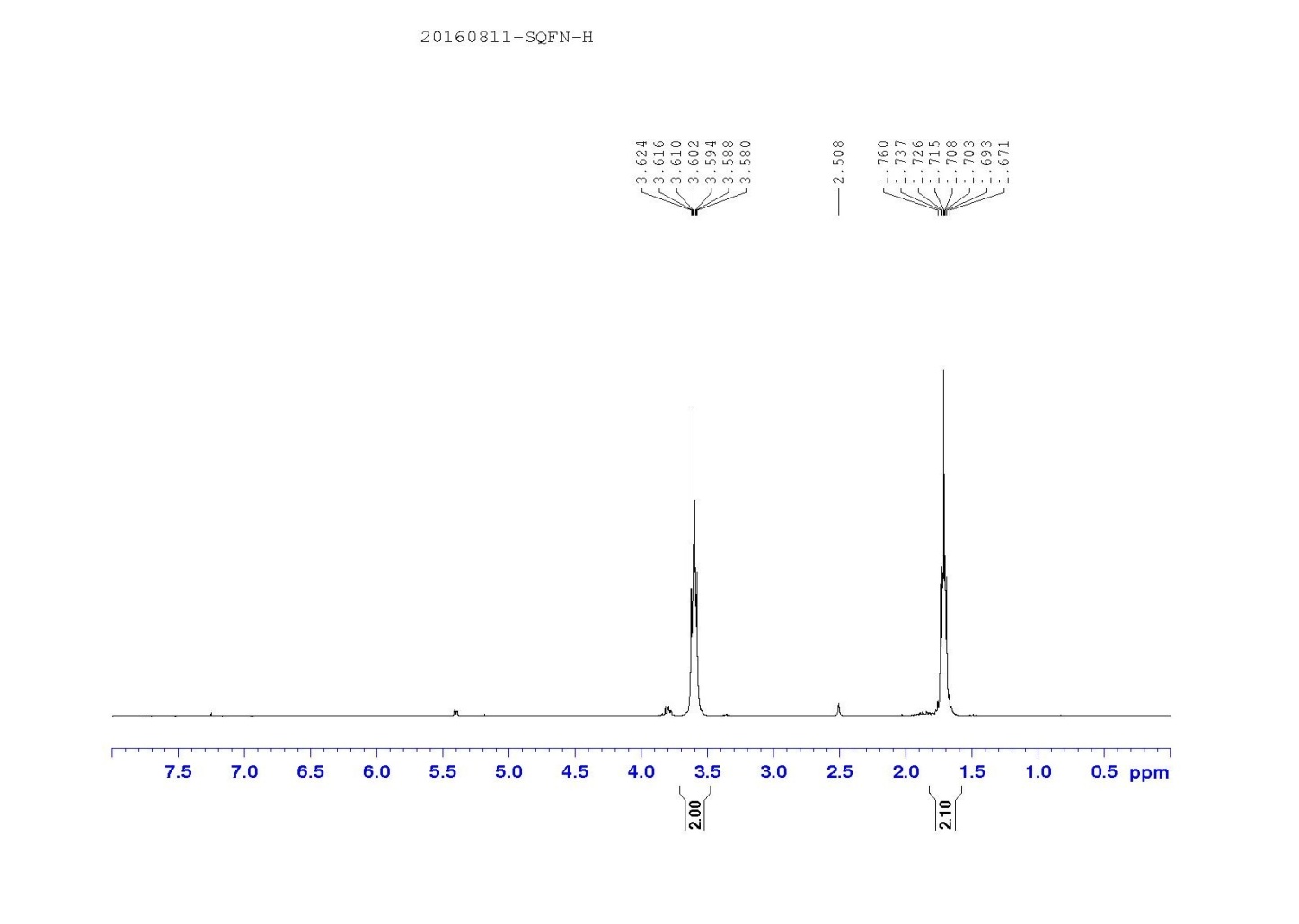


2. ^13^C NMR spectra of product 3a


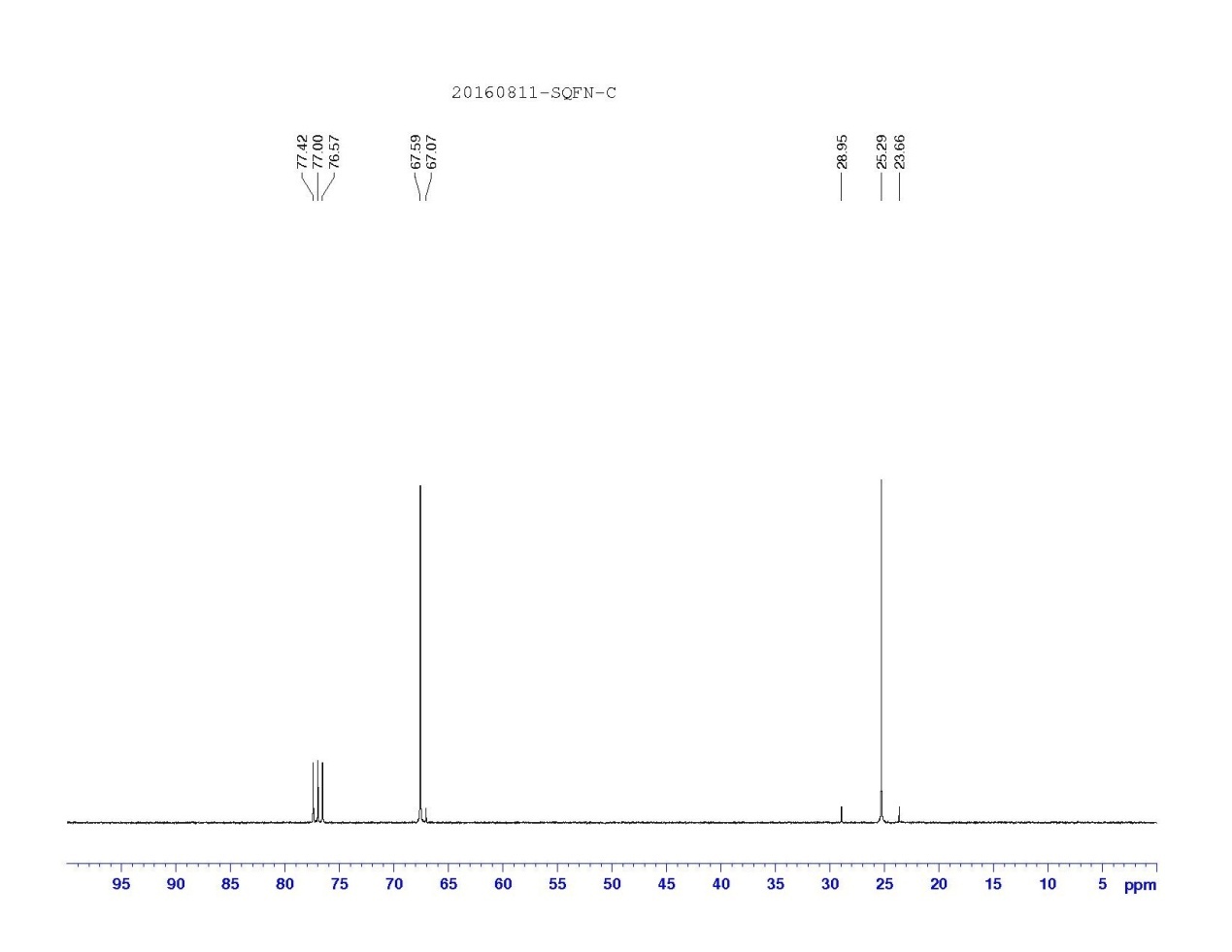


3. MS spectra of product 3a

4. ^1^H NMR spectra of product 3b


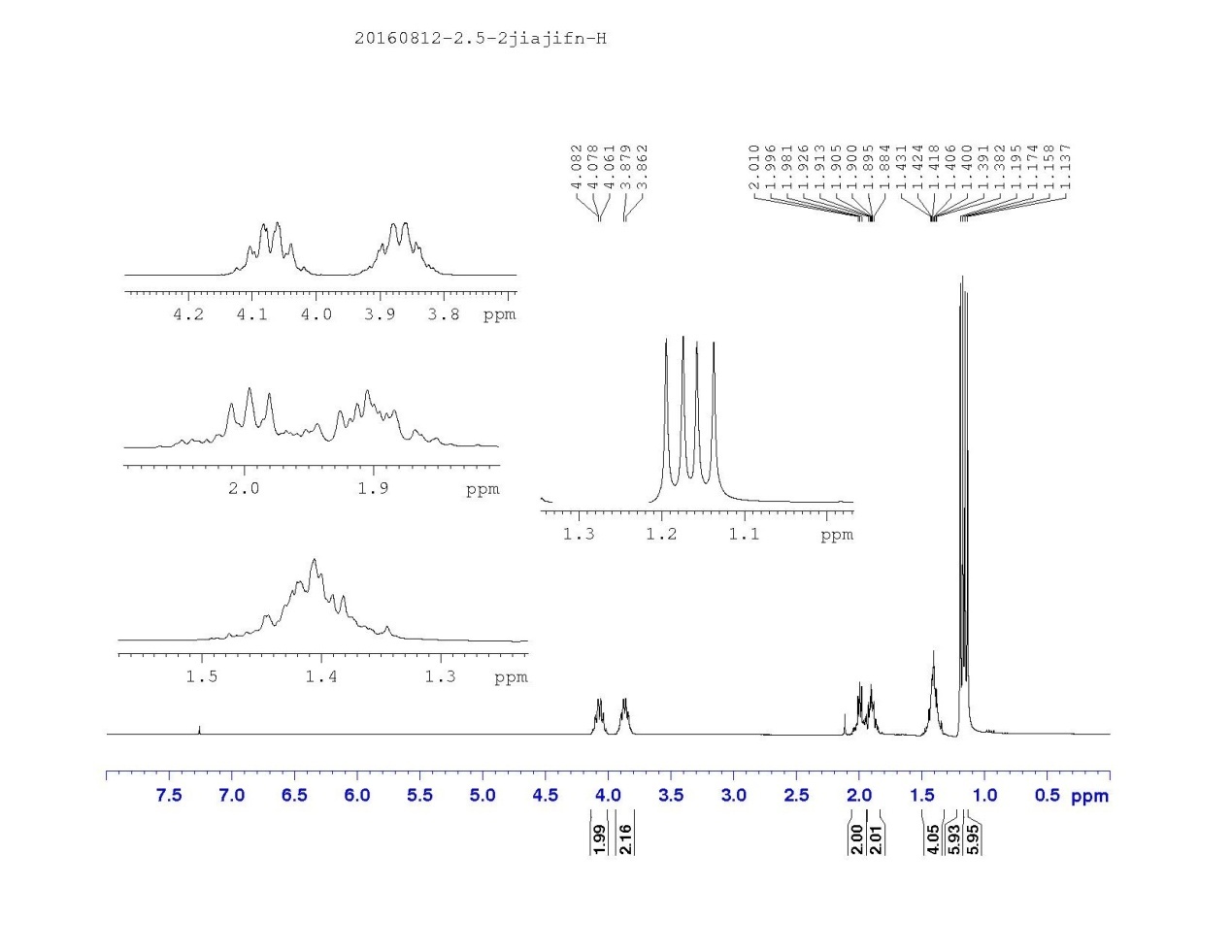


5. ^13^C NMR spectra of product 3b


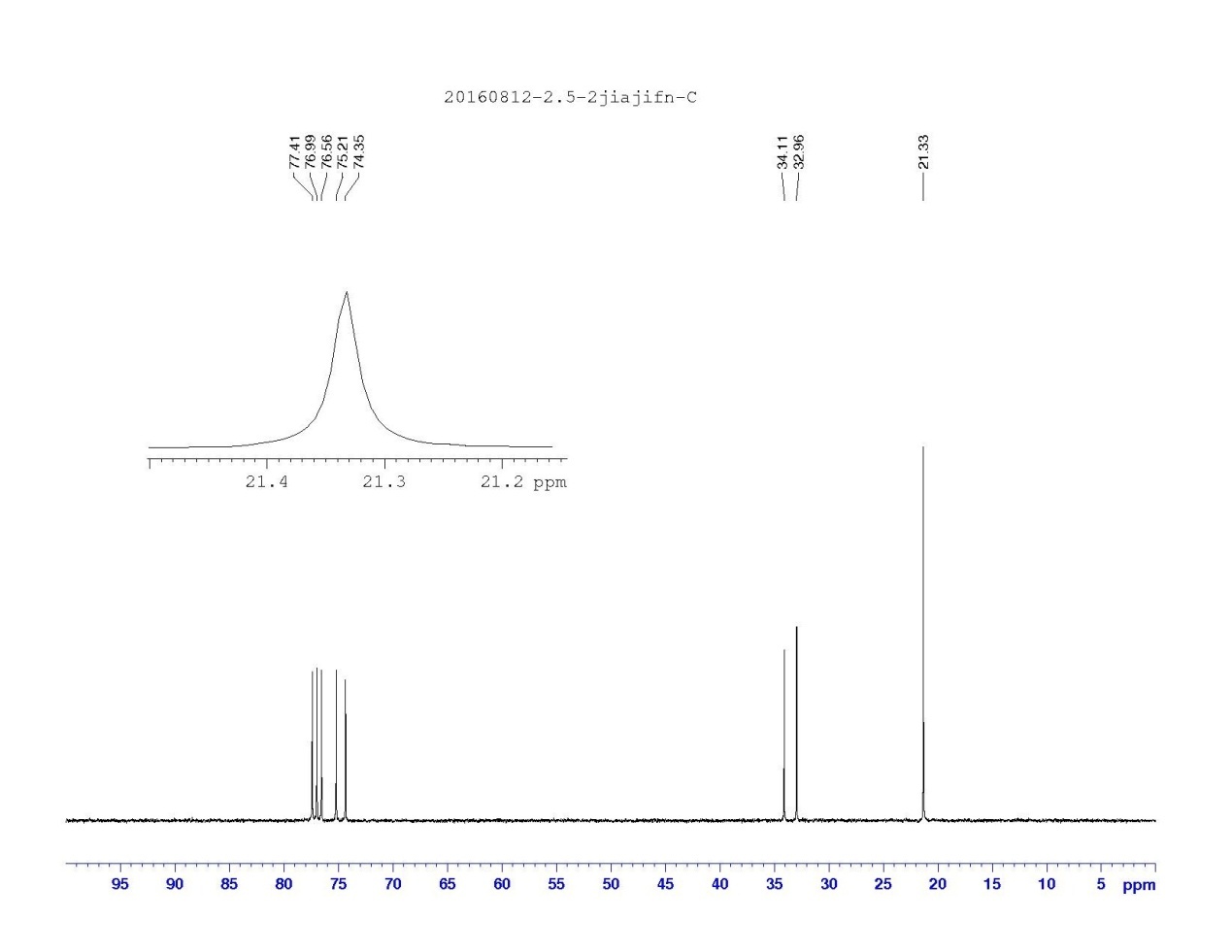


6. MS spectra of product 3b

7. ^1^H NMR spectra of product 3c


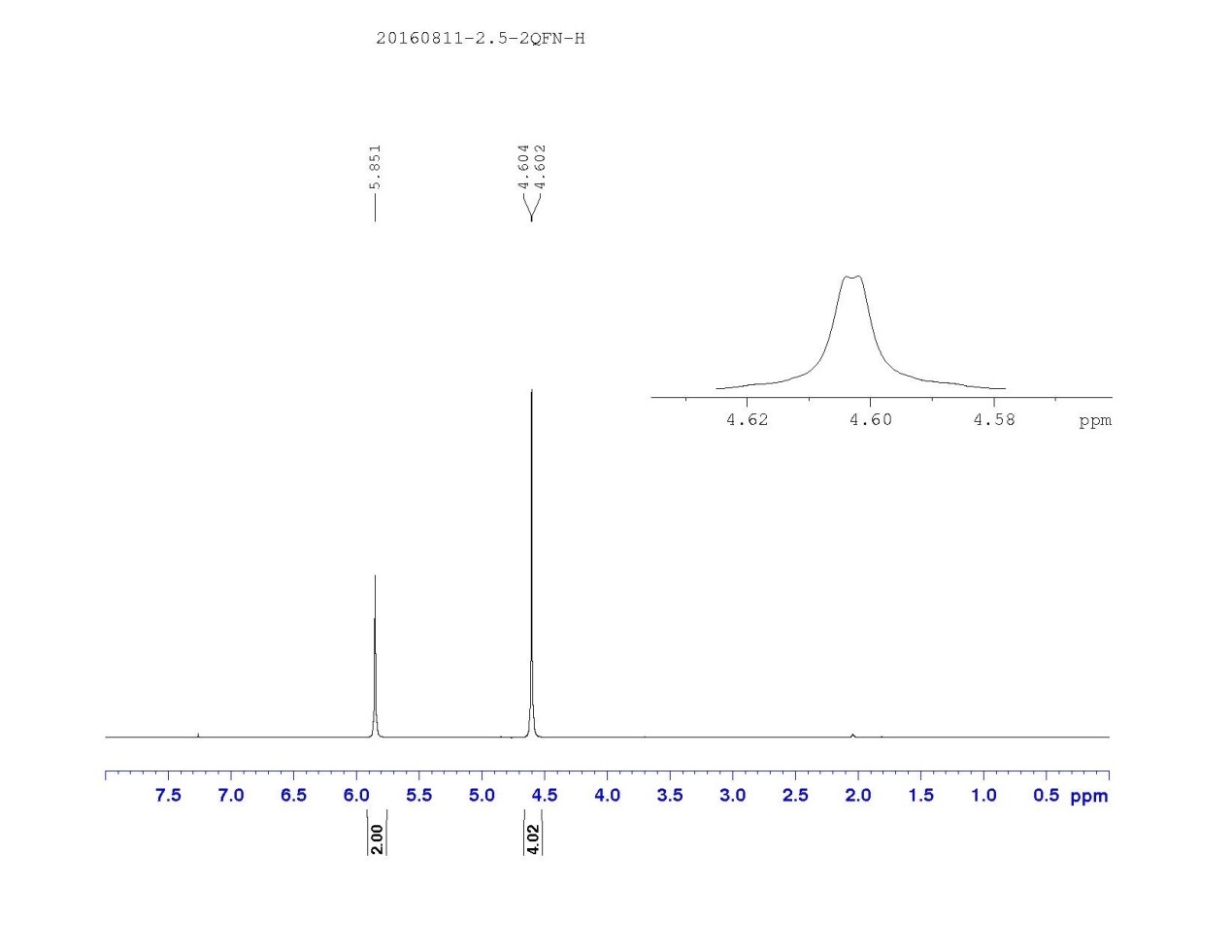


8. ^13^C NMR spectra of product 3c


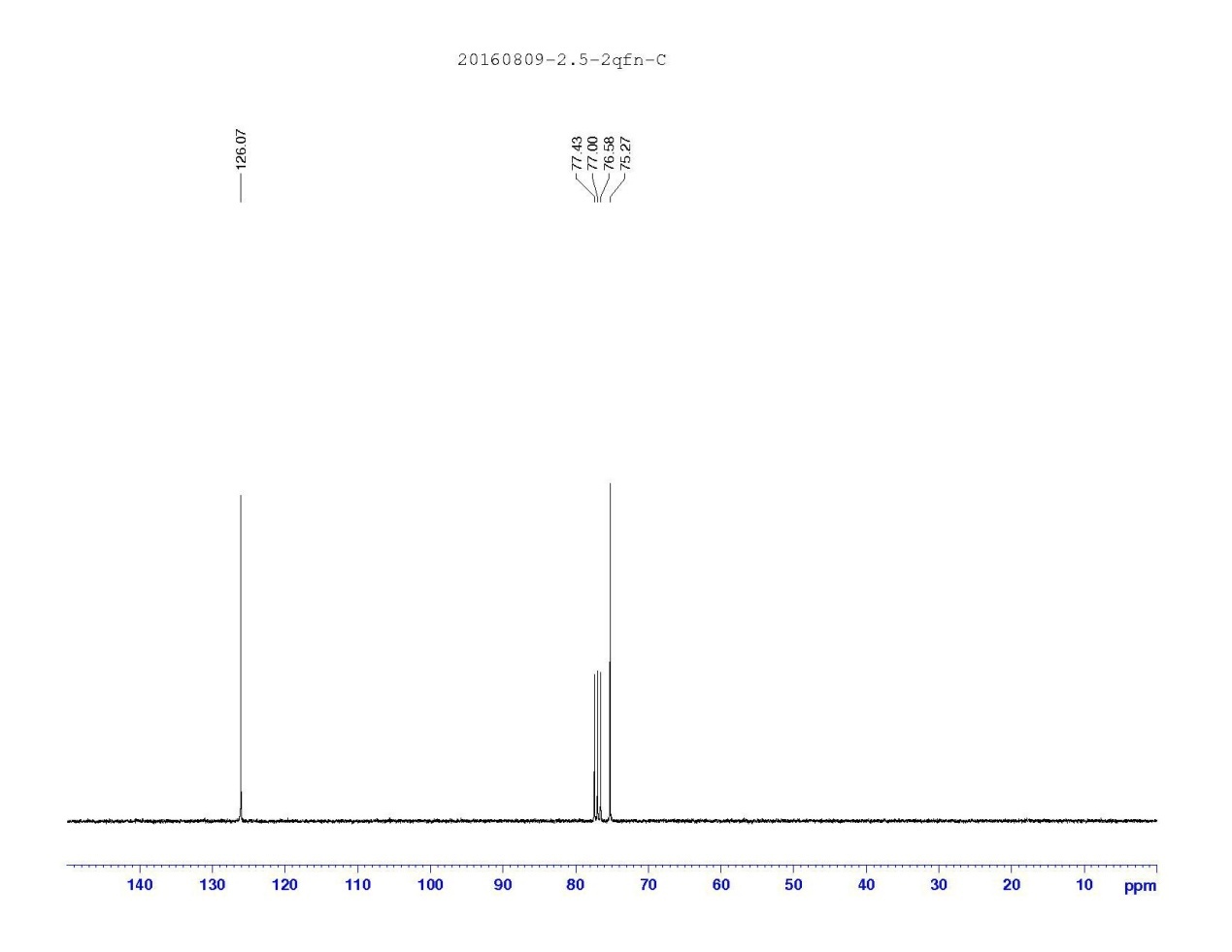


9. MS spectra of product 3c

10. ^1^H NMR spectra of product 3d


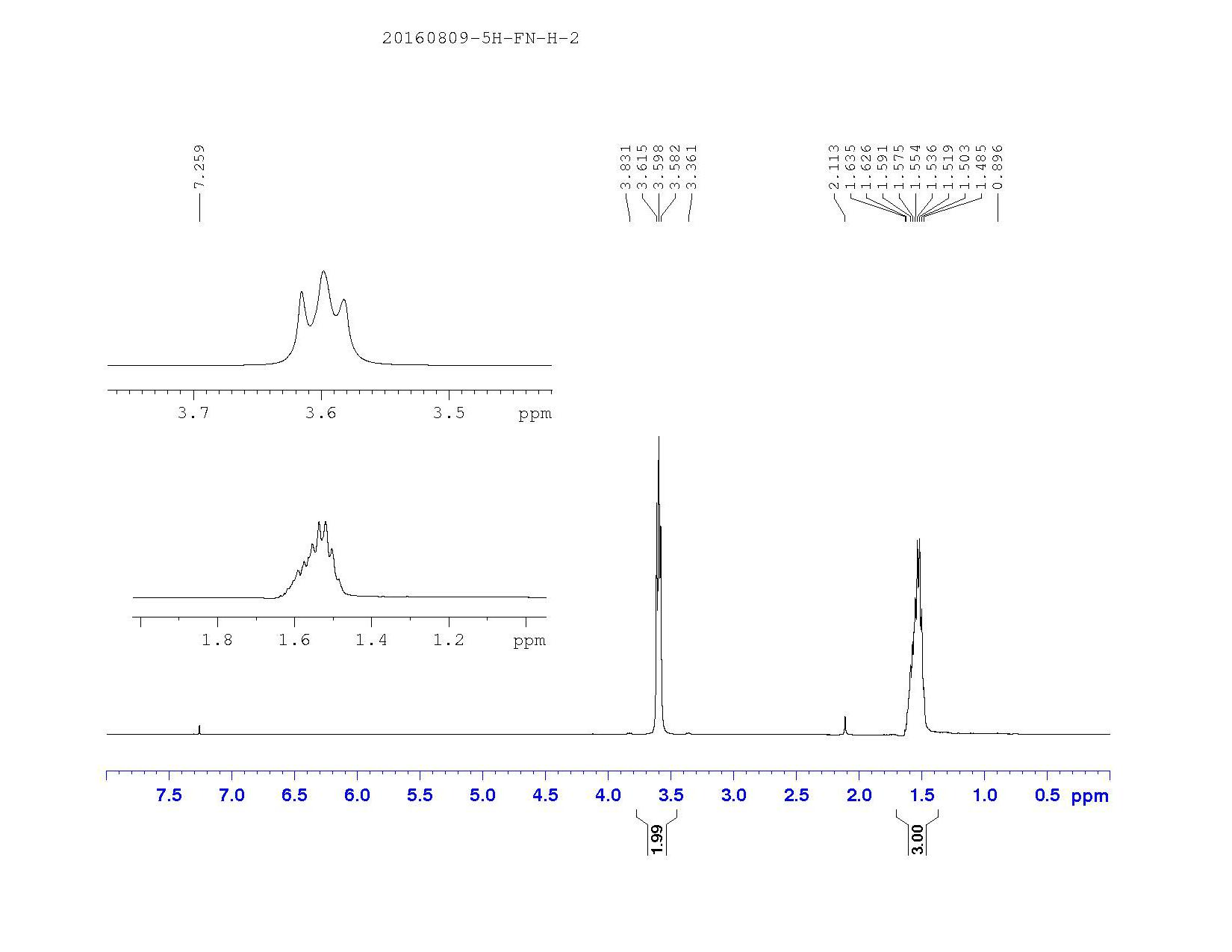


11. ^13^C NMR spectra of product 3d


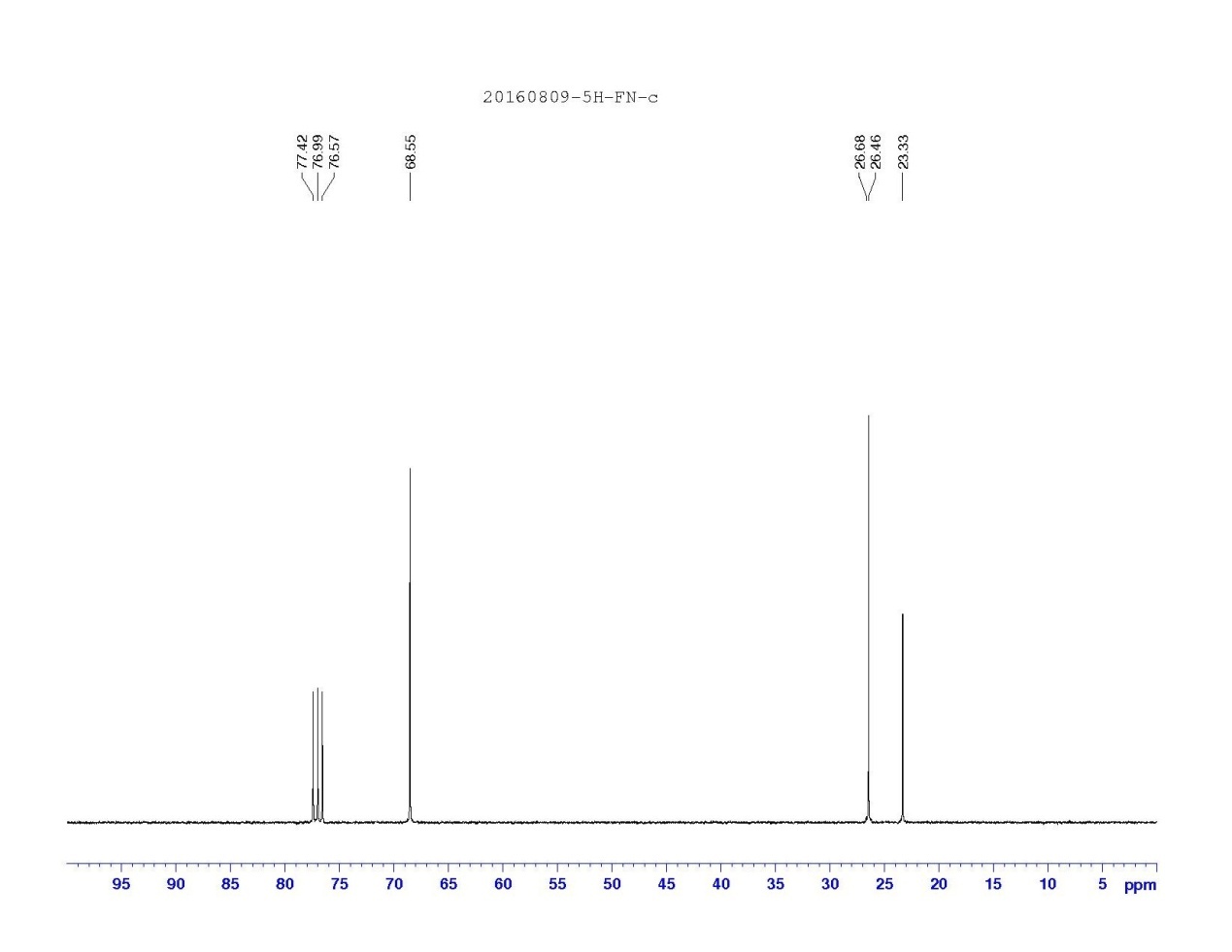


12. MS spectra of product 3d

13. ^1^H NMR spectra of product 3e


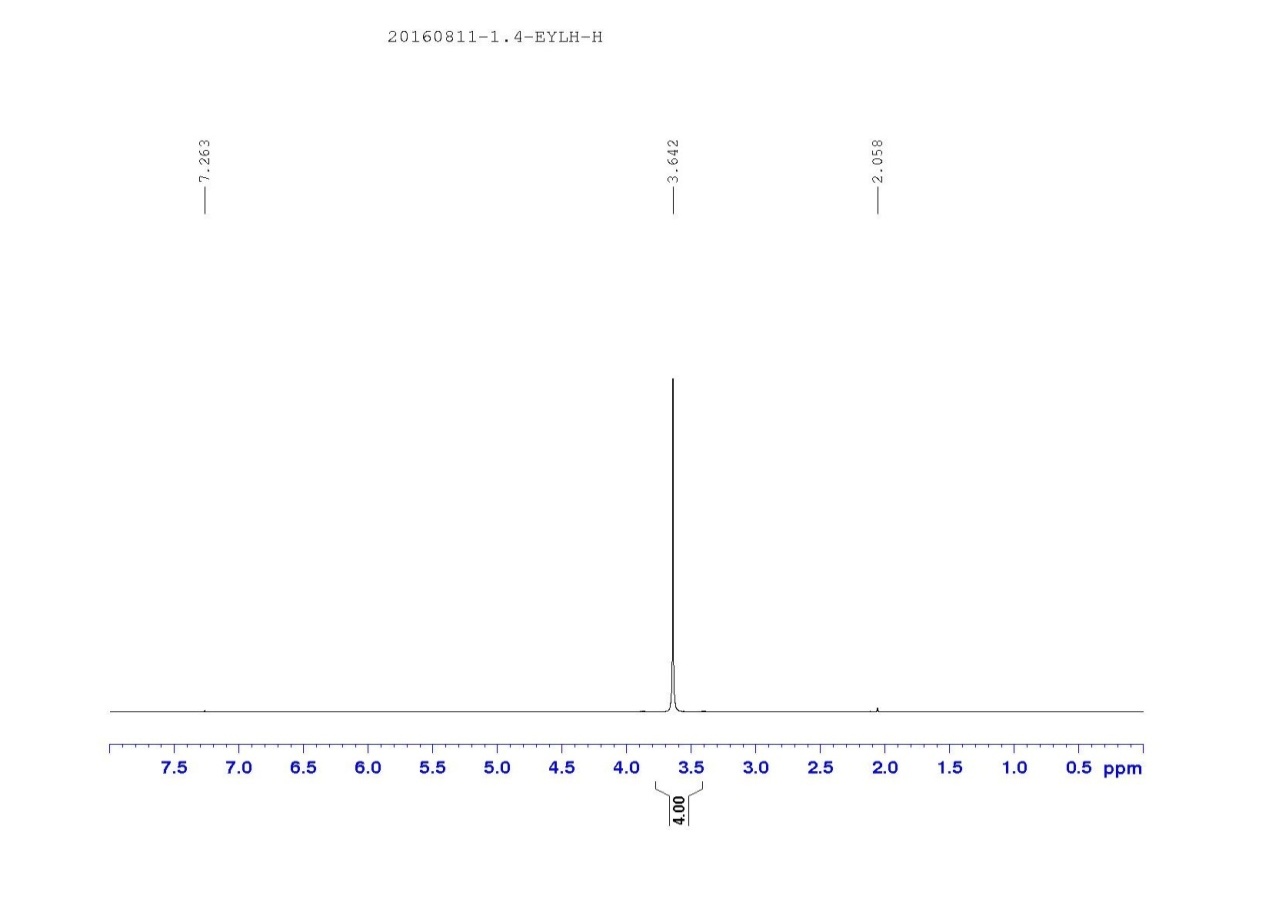


14. ^13^C NMR spectra of product 3e


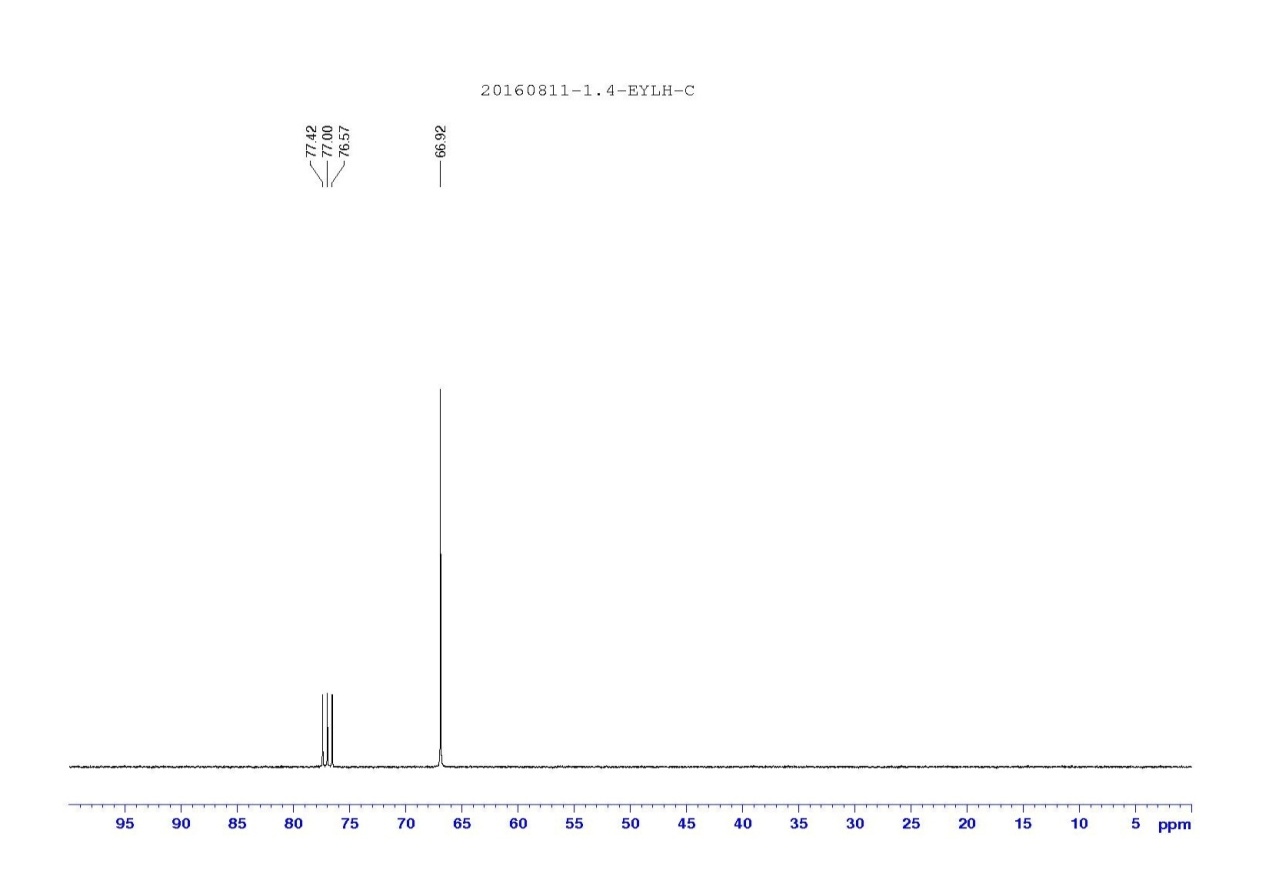


15. MS spectra of product 3e

16. ^1^H NMR spectra of product 3g


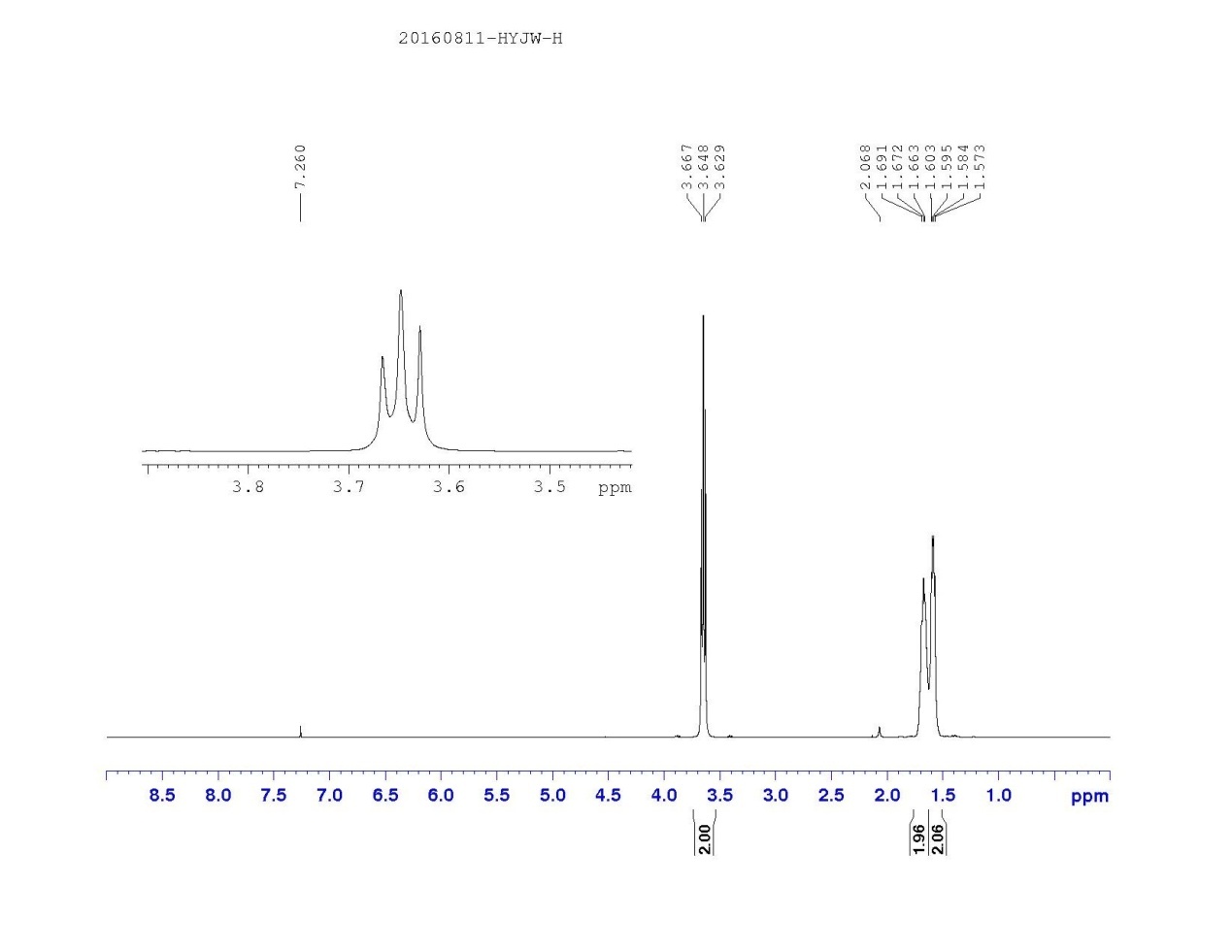


17. ^13^C NMR spectra of product 3g


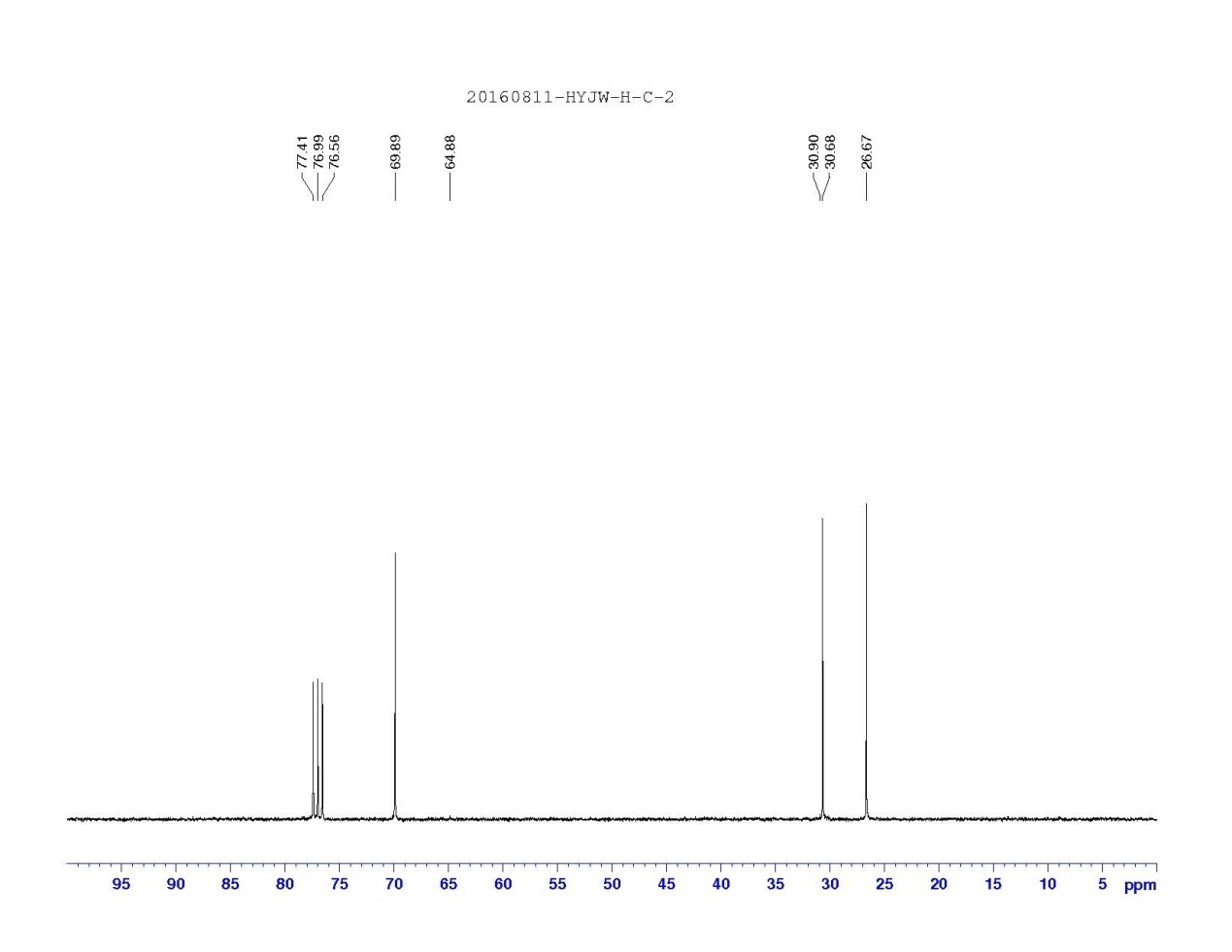


18. MS spectra of product 3g

19. GC spectra of product 3a


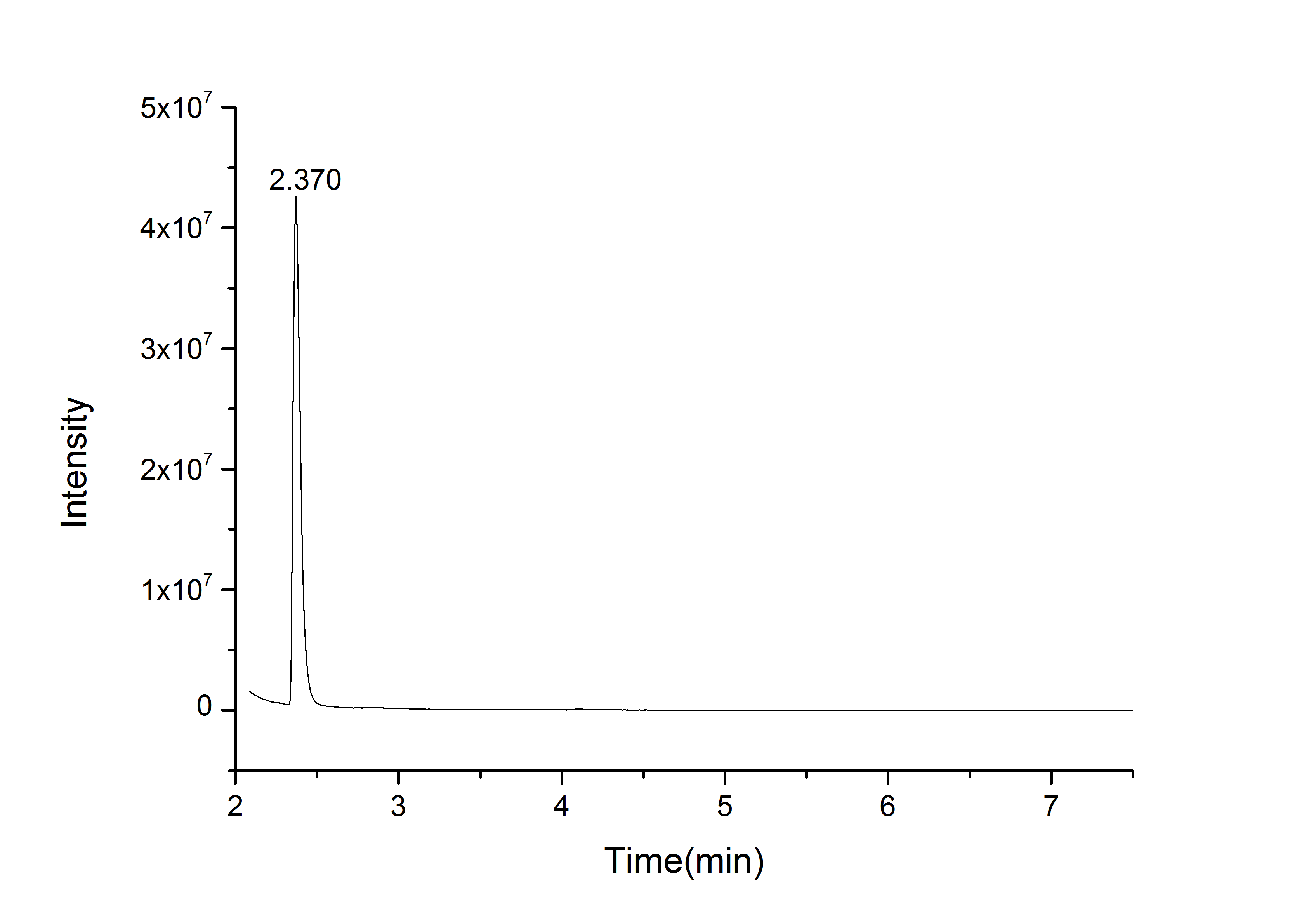


20. GC spectra of product 3b (cis-trans isomerism)


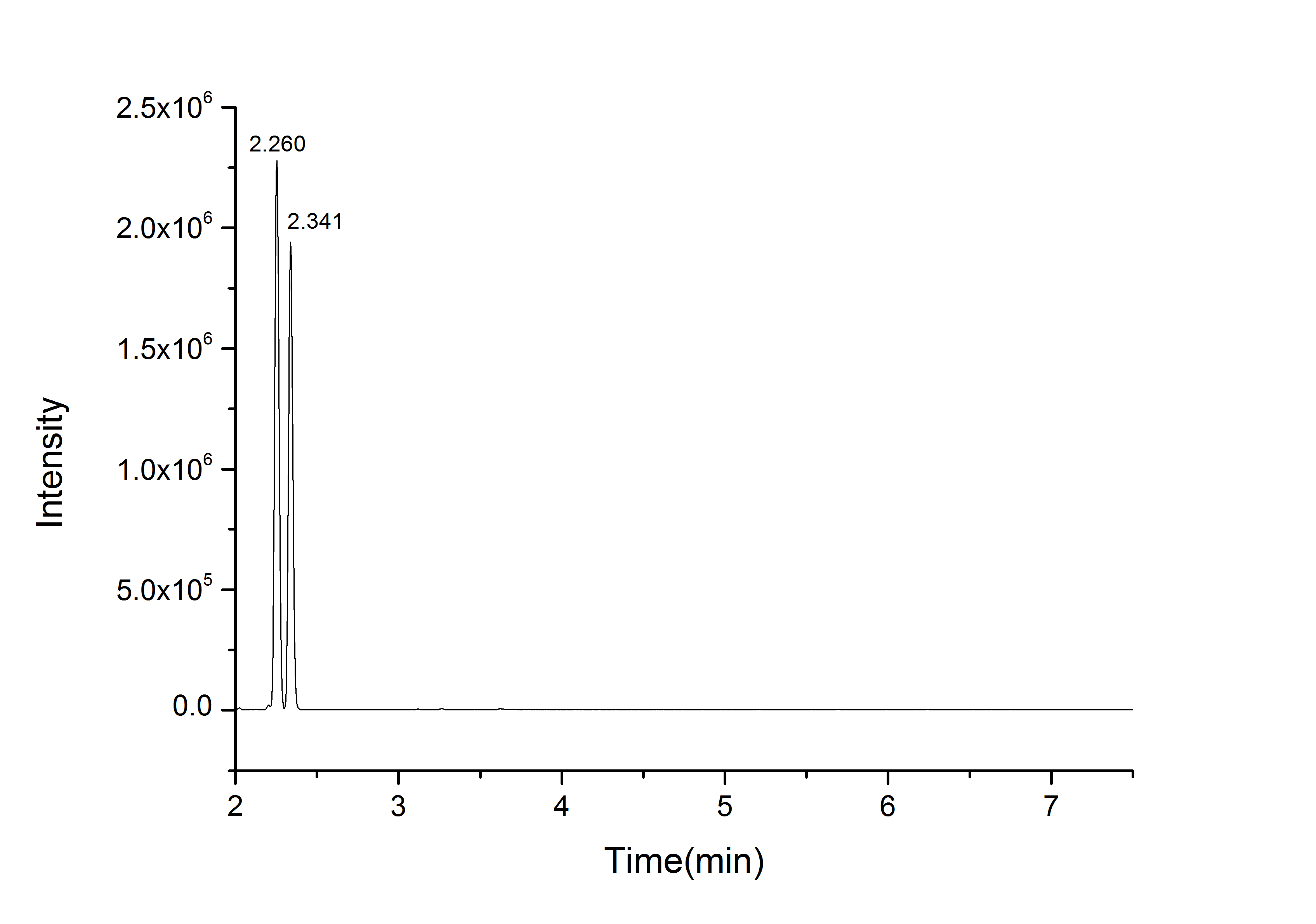


21. GC spectra of product 3c


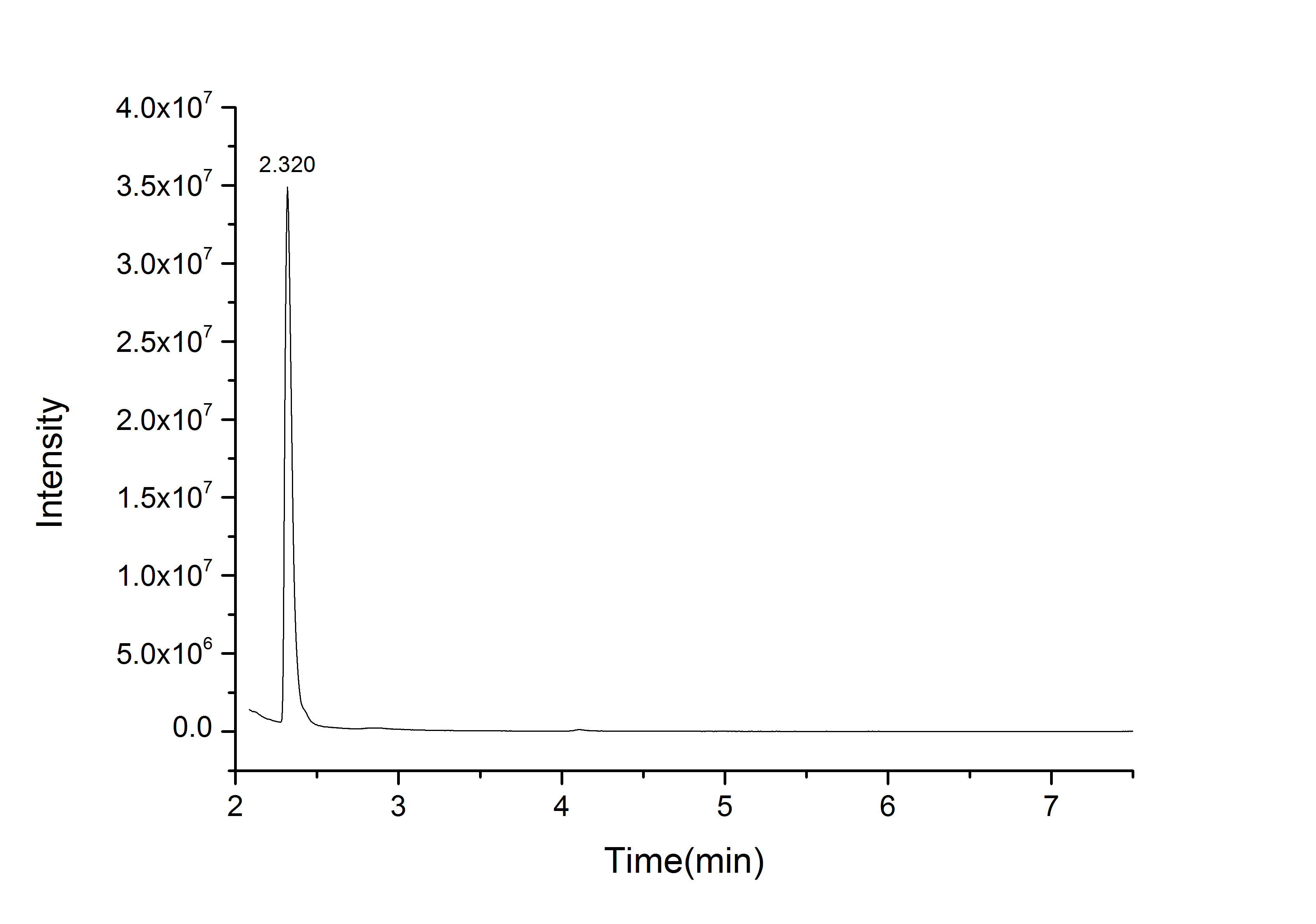


22. GC spectra of product 3d


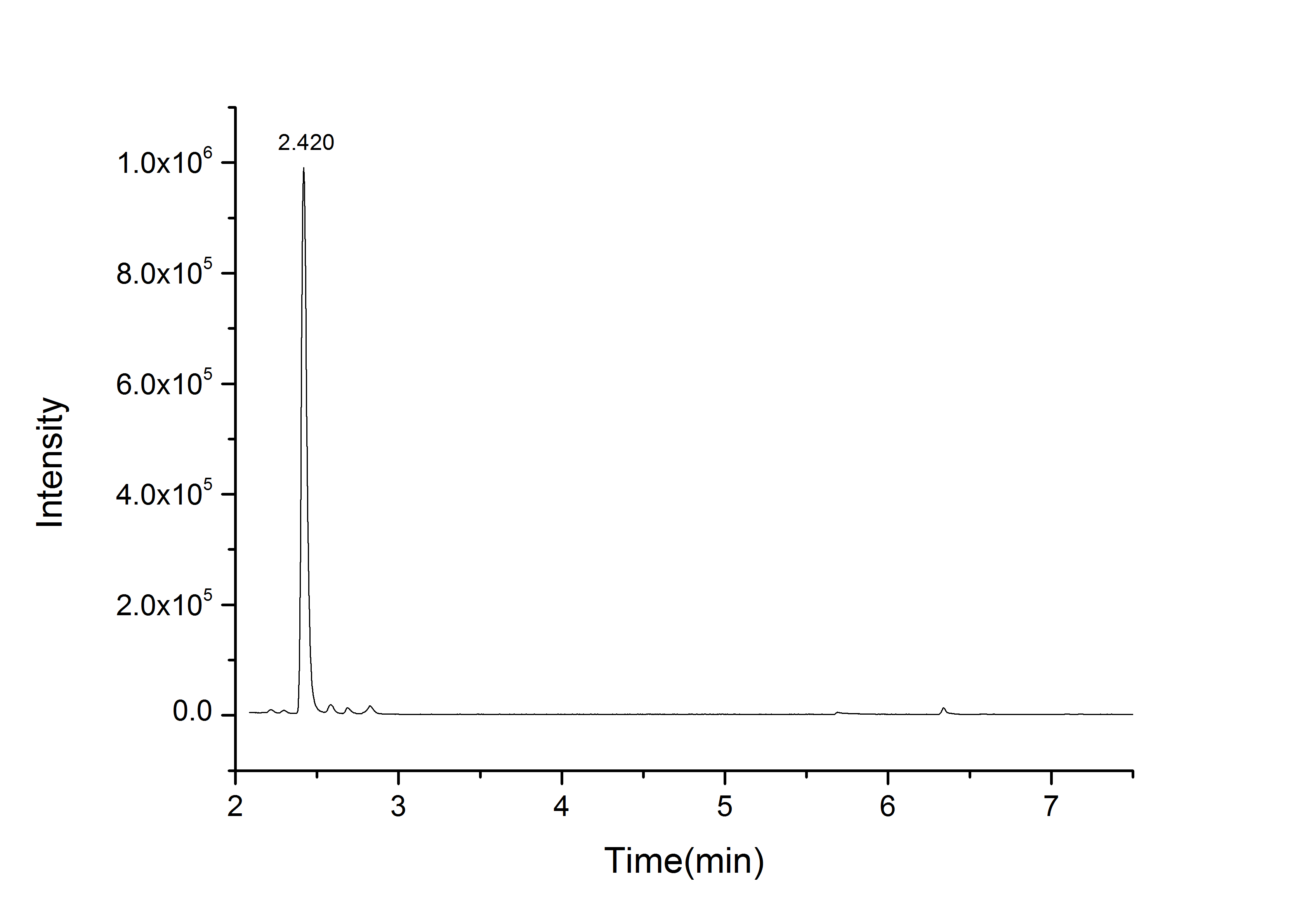


23. GC spectra of product 3e


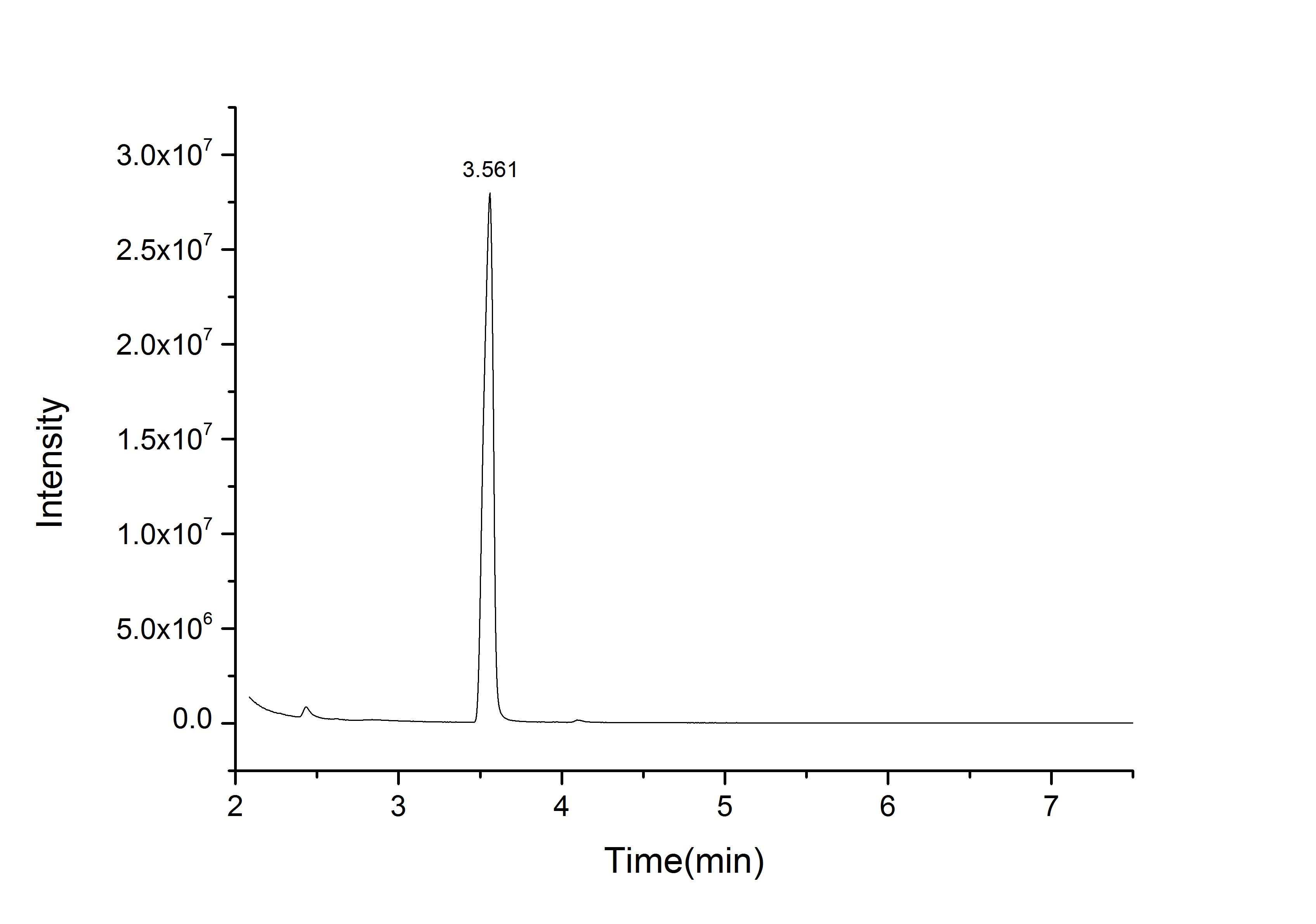


23. GC spectra of product 3g


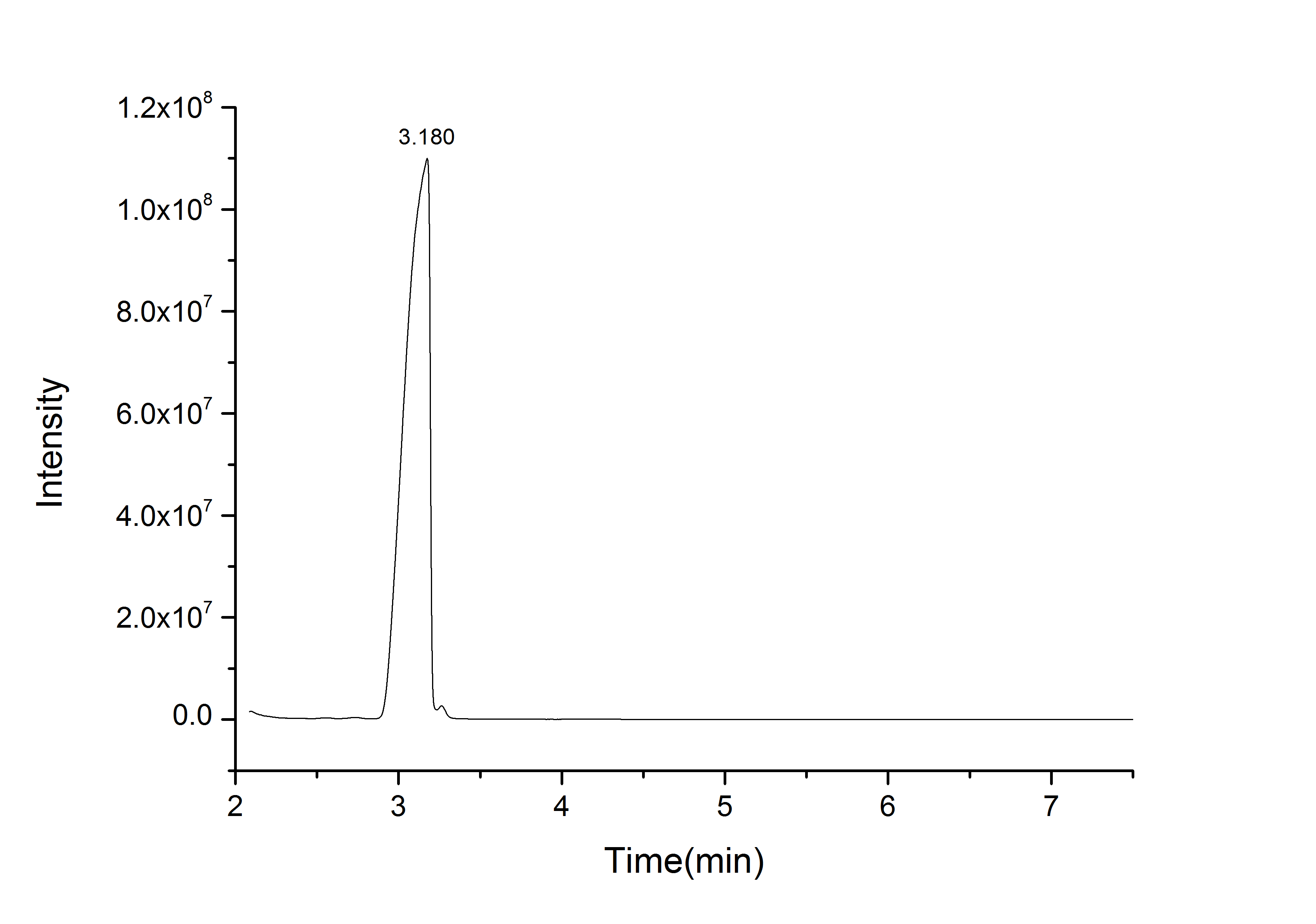

Supplement: 1H NMR, 13C NMR, MS and GC spectras of products 3a–3g [file rsos180740supp1.doc]
